# *Lithobius (Monotarsobius) monoforaminis* sp. n., a new species of lithobiid centipede from central China (Chilopoda, Lithobiomorpha, Lithobiidae)

**DOI:** 10.3897/zookeys.193.2802

**Published:** 2012-05-14

**Authors:** Huiqin Ma, Sujian Pei, Dayong Wu, Hongjun Lin, Yonghua Gai

**Affiliations:** 1 Scientific Research Office, Hengshui University, Hengshui, Hebei 053000, P. R. China; 2 Department of Life Sciences, Hengshui University, Hengshui, Hebei 053000, P. R. China; 3 State Key Laboratory of Palaeobiology and Stratigraphy, Nanjing Institute of Geology and Palaeontology, Chinese Academy of Sciences; Nanjing, Jiangsu 210008, P. R. China

**Keywords:** Lithobiidae, *Lithobius (Monotarsobius) monoforaminis*, China, identification key

## Abstract

The present paper describes a new species *Lithobius (Monotarsobius) monoforaminis* **sp. n.** (Lithobiomorpha: Lithobiidae) recently discovered from Shaanxi and Shanxi provinces, Central China. Morphologically it resembles *Lithobius (Monotarsobius) minimus* Farzalieva, 2006 from Eastern Kazakhstan, but could be well distinguished from the latter having only one pore on the coxae of legs 12–15 and different plectrotaxy, and by lacking a wart on the male tibia 15. A key to the Chinese *Lithobius (Monotarsobius)* species is presented.

## Introduction

The centipede subgenus *Lithobius (Monotarsobius)* Verhoeff, 1905 (Lithobiomorpha: Lithobiidae) comprises 114 species known from Asia, Europe, and North Africa (Pocock 1895; Trotzina 1895; Attems 1901, 1904; Dobroruka 1960, 1979; Zalesskaja 1978; Farzalieva and Zalesskaja 2002; Farzalieva 2006; Zapparoli 2006; Zapparoli and Edgecombe 2011; Dányi and Tuf 2012). It is characterized by the presence of fused tarsi of legs 1–13 and antennal articles fixed at 20 or thereabouts (Eason 1992).

Sixty-seven species and subspecies of Lithobiomorpha are hitherto known from China (Attems 1938, 1953; Takakuwa 1939, 1940, 1941, 1942; Takakuwa and Takashima 1949; Chamberlin and Wang 1952; Wang 1959, 1963; Zalesskaja 1978; Wang and Mauriès 1996; Zhang 1996; Eason 1992, 1997; Chao 2005; Zapparoli 2006; Ma et al. 2007a, 2007b, 2008a, 2008b, 2009; Pei et al. 2011), of which only nine belong to subgenus *Monotarsobius*. Herewith we describe a new species of *Monotarsobius* which was recently found in the Shaanxi and Shanxi provinces. This is the first reliable record of the subgenus in this region of China.

## Methods

All specimens were hand-collected under leaf litter or stones. The material was examined with the aid of a Motic-C microscope, made in China. Colour description is based on specimens in 75% alcohol, and body length is measured from anterior margin of the cephalic plate to posterior end of postpedal tergite. Type specimens are deposited in the College of Life Sciences, Hebei University, Baoding, China. Terminology for external anatomy follows Bonato et al. (2010).

The following abbreviations are used in the text and tables: T, TT = tergite, tergites; S, SS = sternite, sternites; C = coxa, t = trochanter, P = prefemur, F = femur, Ti = tibia, a = anterior, m = median, p = posterior.

## Taxonomy

### Lithobiidae Newport, 1844

#### Lithobius (Monotarsobius) monoforaminis sp. n.

urn:lsid:zoobank.org:pub:A8F0E269-29BB-4C6F-B575-3FA298B01225

http://species-id.net/wiki/Lithobius_monoforaminis

##### Material examined.

**Figures 1–6.**
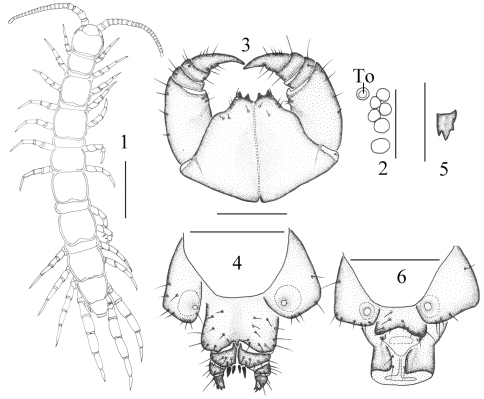
*Lithobius (Monotarsobius) monoforaminis* sp.n., **1–3** holotype, male **1** habitus, dorsal view, scale 1 mm **2** ocelli and Tömösváry’s organ (To), lateral view, scale 250 µm **3** forcipular segment, ventral view, scale 500 µm **4–5** paratype, female **4** posterior segments and gonopods, ventral view, scale 500 µm **5** right gonopod, ventral view, scale 250 µm **6** holotype, male: posterior segments and gonopods, ventral view, scale 500 µm.

**Holotype.** ♂ (Fig. 1), body length 7.7 mm, cephalic plate 0.76 mm long, 0.76 mm broad, Huashan Mountain, Huayin County, Xian City, Shaanxi Province, 34°31'N, 110°05'E, 438 m, 10 October 2005, leg. Yong-hua Gai, Hui-qin Ma.

**Paratypes.** 5 ♀♀, 3 ♂♂, same data as holotype.

##### Other materials.

13 ♀♀, 12 ♂♂, Yongji County, Yuncheng City, Shanxi Province, 34°51'N, 110°29'E, 388 m, 8 October 2005, leg. Yong-hua Gai, Hui-qin Ma.

##### Etymology.

The specific name refers to the presence of a single pore on the coxae of legs 12–15.

##### Diagnosis.

A *Lithobius (Monotarsobius)* species with antennae composed of 16–22 articles, commonly 20 articles; 6–7 ocelli on each side, arranged in two irregular rows, the two posterior ocelli comparatively large; Tömösváry’s organ moderately small, slightly smaller than adjoining ocelli, or equal to adjoining ocelli; 2+2 coxosternal teeth; porodonts moderately slender, posterolateral to lateral tooth; posterior angles of all tergites without triangular projections; coxal pores 1111, round; female gonopods with 2+2 moderately small, coniform spurs; terminal claw tridentate; male gonopods short and small, with 1–2 long setae on the terminal segment.

##### Description.

Body length: 6.9–8.6 mm, cephalic plate 0.75–0.96 mm long, 0.75–0.96 mm wide.

Colour: basal antennal articles pale yellow-brown to chocolate, transition to yellow brownish from the twelfth article onwards, distalmost one yellow-brownish; tergites yellow-brown; cephalic plate, TT 1, 14 and 15 pale yellow-brown; pleural region pale grey with a yellowish hue, SS pale grey to grey with a purple hue; distal part of forcipules brown, basal and proximal parts of forcipules, forcipular coxosternite and SS 14 and 15 pale yellow-brown; all legs pale purple to grey, basal tarsus pale purple, distal tarsus yellow-brown.

Antennae: composed of 16–22 articles, commonly 20+20 articles; basal article long about equal to wide, the second markedly longer than wide, following articles gradually shortening, distal article much longer than wide, up to 2.0–2.5 times as long as wide; abundant setae on the antennal surface, less so on the basal articles, gradually increase in density towards the fourth article, then more or less constant in number.

Cephalic plate: smooth, convex, pigment concentrated as close netlike vein, long equal to wide; tiny setae emerging from pores and long setae scattered sparsely over the whole surface; frontal marginal ridge with shallow anterior median furrow; posterior margin of cephalic plate straight (Fig. 1).

Ocelli: six–seven oval to rounded on each side (Fig. 2) situated in two irregular rows; terminal two comparatively large, other ocelli about equal in size; ocelli domed, translucent, usually darkly pigmented.

Tömösváry’s organ: comparatively small (Fig. 2-To), nearly rounded; situated at anterolateral margin of cephalic plate, slightly smaller than the adjoining ocelli or equal in size.

Forcipular coxosternite: subtrapezoidal (Fig. 3), anterior margin narrow, external side lightly longer than internal side; median diastema moderately deep, V-shaped; anterior margin with 2+2 teeth; porodonts comparatively sharp, lying posterolateral to the lateral tooth (Fig. 3); some scattered setae on the ventral side of coxosternite.

Tergites: smooth, without wrinkles, backside slightly hunched; T 1 posterolaterally narrower than anterolaterally, generally trapeziform, narrower than T 3 and the cephalic plate, T3 slightly narrower than the cephalic plate; posterior margin of T 1 slightly convex or straight, posterior margin of T 3 straight, posterior margin of TT 5, 8, 10, 12 and 14 slightly concave, posterior margin ridge of TT 3, 5, 8, 10 and 12 continuous; all posterior angles generally rounded, without triangular projections; lateral margin ridge of all tergites continuous; tiny setae scattered very sparsely over the surface.

Sternites: narrower posteriorly, generally trapeziform, comparatively smooth, setae emerging from pores scattered very sparsely over the surface, 1–3 slightly long setae on the surface of the anterior part of each sternite, 1–2 slightly long setae on the surface of the posterior part of each sternite.

Legs: strong, tarsi fused on legs 1–13, well-defined on legs 14 and 15; all legs with claws, fairly long, curved ventrad; anterior and posterior accessory spines on legs 1–14, the anterior one moderately slender, forming a small angle with the claw, the posterior spine short and strong, forming a large angle with the claw; no accessory spines on legs 15. Short to comparatively long setae scattered very sparsely over the surface of all segments of all legs, more setae scattered on the surface of tarsus; legs 14 and 15 thicker and stronger than other legs in the male; tarsus 1 4.4–5.5 times as long as wide, tarsus 2 about 73.3%–95.7% length of tarsus on legs 15. Plectrotaxy as in Table 1.

**Table 1.** Plectrotaxy of *Lithobius (Monotarsobius) monoforaminis* sp. n.

**legs**	**ventral**					**dorsal**				
	**C**	**t**	**P**	**F**	**Ti**	**C**	**t**	**P**	**F**	**Ti**
1			p	am	m			p	ap	a
2–10			p	am	m			p	ap	ap
11			p	am	m			p	ap	ap
12			p	am	m			mp	p	p
13			mp	m	am			mp	p	p
14		m	mp	m		a		mp		
15		m	mp	m		a		mp		

Coxal pores: 1111, round; coxal pore field set in a relatively shallow groove, the fringe of coxal pore-field with slightly eminence.

Female S 15: generally trapeziform, anterior half being broader posterior margin straight, long setae scattered sparsely over the surface; the sternite of genital segment well chitinised, wider than long; posterior margin of genital sternite deeply concave between the condyles of gonopods, except for a small, median approximately fingerlike bulge; short to long setae sparsely scattered over the ventral surface of the genital segment. Female gonopod: first article fairly broad, bearing 6–8 long setae, arranged in 3 irregular rows; 2+2 moderately small, coniform spurs, inner spur smaller (Fig. 4); second article with 3–4 rather long setae arranged in 2 irregular rows on its ventral side and two stout dorsolateral setae; third article usually with 2–3 long setae on its ventral surface and one stout dorsolateral seta; terminal claw tridentate, outer and inner denticles smaller than the middle one (Fig. 5).

Male S 15: trapeziform, the anterior half being broader; posterior margin straight, long setae scattered sparsely over the surface. Male first genital sternite: wider than long, usually well chitinized; posterior margin quite deeply concave between the gonopods, no bulge medially; comparatively long setae evenly scattered on the ventral surface, few setae near the S 15; gonopods short and small, with 1–2 long setae, apically slightly chitinized (Fig. 6).

**Habitat preferences.** The specimens were collected under stones and in leaf litter in a Larix forest.

**Remarks.** *Lithobius (Monotarsobius) monoforaminis* sp. n. is morphologically close to *Lithobius (Monotarsobius) minimus* Farzalieva, 2006 from Eastern Kazakhstan, with which it shares the following mutual characters: terminal claw of female gonopods tridentate, antennae commonly of 20 articles and two stout dorsolateral setae on the second article of female gonopod. The new species can be readily could be readily distinguished from the latter by having male tibia 15 without a distodorsal, elongate wart with a crater at apex and coxal pores and the 15^th^ ventral plectrotaxy 01210 (vs. 01320). *Lithobius (Monotarsobius) monoforaminis* sp. n. differs from *Lithobius (Monotarsobius) ketmenensis* Farzalieva, 2006 and *Lithobius (Monotarsobius) amplinus* Farzalieva, 2006 by having fewer ocelli and coxal pores, different plectrotaxy and lacking modifications on male tibia 15.

### Key to the Chinese species of *Lithobius (Monotarsobius)*

To assist in the identification of the Chinese species of *Lithobius (Monotarsobius)*, the following key is offered. This key emphasizes characters that can be examined without high-magnification microscopy; moreover, these characters are specific to the taxa occurring in China.

**Table:** 

1	Four ocelli on each side of cephalic plate, 17+17 antennal articles	*Lithobius (Monotarsobius) crassus* (Loksa, 1965)
–	Five or more ocelli on each side of cephalic plate, not less than 18+18 antennal articles	2
2	Five ocelli on each side of cephalic plate	*Lithobius (Monotarsobius) alticus* (Loksa, 1965)
–	Six or more ocelli on each side of cephalic plate	3
3	Second article of female gonopod with dorsolateral setae	4
–	Second article of female gonopod without dorsolateral setae	6
4	Second article of female gonopod with three dorsolateral setae, 2222–3443 coxal pores	*Lithobius (Monotarsobius) crassipes* L. Koch, 1862
–	Second article of female gonopod with two dorsolateral setae, 1111–2222 coxal pores	5
5	1222–2222 coxal pores	*Lithobius (Monotarsobius) ferganensis* (Trotzina, 1894)
–	1111 coxal pores	*Lithobius (Monotarsobius) monoforaminis* sp. n.
6	Terminal claw of the female gonopod simple	*Lithobius (Monotarsobius) ramulosus* (Takakuwa, 1941)
–	Terminal claw of the female gonopod bidentate or tridentate	7
7	Terminal claw of female gonopod tridentate	8
–	Terminal claw of female gonopod bidentate	9
8	Tömösváry’s organ slightly smaller than adjoining ocellus; terminal ocellus largest	*Lithobius (Monotarsobius) songi* Pei, Ma, Shi, Wu, Zhou, 2011
–	Tömösváry’s organ slightly larger than adjoining ocellus or about same in size; terminal two ocelli largest	*Lithobius (Monotarsobius) subspinipes* Ma, Pei, Zhu, Zhang, Liu, 2009
9	Tömösváry’s organ larger than largest ocellus, antennae 20–25 articles	*Lithobius (Monotarsobius) holstii* (Pocock, 1895)
–	Tömösváry’s organ about same size as the adjoining ocelli, antennae 19 articles	*Lithobius (Monotarsobius) obtusus* (Takakuwa, 1941)

## Supplementary Material

XML Treatment for Lithobius (Monotarsobius) monoforaminis
